# New insights into the functional role of retrotransposon dynamics in mammalian somatic cells

**DOI:** 10.1007/s00018-021-03851-5

**Published:** 2021-05-14

**Authors:** Arianna Mangiavacchi, Peng Liu, Francesco Della Valle, Valerio Orlando

**Affiliations:** Biological Environmental Science and Engineering Division, King Abdullah University of Science and Technology (KAUST), Thuwal, Saudi Arabia

**Keywords:** Retrotransposon, Repetitive RNA, Cell identity, Gene expression, Development

## Abstract

Retrotransposons are genetic elements present across all eukaryotic genomes. While their role in evolution is considered as a potentially beneficial natural source of genetic variation, their activity is classically considered detrimental due to their potentially harmful effects on genome stability. However, studies are increasingly shedding light on the regulatory function and beneficial role of somatic retroelement reactivation in non-pathological contexts. Here, we review recent findings unveiling the regulatory potential of retrotransposons, including their role in noncoding RNA transcription, as modulators of mammalian transcriptional and epigenome landscapes. We also discuss technical challenges in deciphering the multifaceted activity of retrotransposable elements, highlighting an unforeseen central role of this neglected portion of the genome both in early development and in adult life.

## Introduction

Retrotransposons, which account for more than 40% of the human and mouse genomes, propagate themselves through transcription and reverse-transcription machinery based on a "copy-and-paste" mechanism [1, 2]. This class of transposable elements (TE) is divided into different orders [3]. *LTR* (*Long Terminal Repeats*) retrotransposons comprise about 8% and 10% of the human and mouse genomes, respectively [4, 5]. *LTR*s are autonomous elements only active in mice and other species [6], not humans [7]. A typical full-length *LTR* has LTRs at both ends and a central region carrying three retroviral open reading frames (ORFs): *gag*, *pol*, and truncated/mutated *Δenv*. Mouse and human *LTR*s can be further classified into several super families that include *endogenous retroviruses* (*ERV*) [3].

Among non-LTR retrotransposons, which are more abundant (17–20%) than *LTR*s in both human and mouse genomes [8], *long interspersed nuclear elements* (*LINE*s) are the major order. These autonomous transposable elements contain two ORFs: ORF1, and ORF2. ORF1 and ORF2 are both required for retrotransposition. ORF1 encodes a ~ 40-kDa protein with nucleic acid-binding and chaperone activity. ORF2 encodes a ~ 150-kDa protein with endonuclease and reverse-transcriptase activity [9–11]. A complete retrotransposition event initiates with the transcription of a full-length *LINE-1* RNA. The *L1* transcript is then exported to the cytoplasm and translated into ORF1p and ORF2p. The binding of the two proteins to the *L1* RNA produces a ribonucleoprotein (RNP) complex, which is imported back into the nucleus. Here, ORF2p uses a free 3′–OH produced through its endonuclease activity on the genomic DNA strand as a primer to reverse transcribe *L1* RNA. Then, *L1* RNA is removed from the intermediate DNA:RNA hybrid, and a second DNA strand is synthesized by ORF2 to generate a novel *L1* insertion. This molecular process is known as target-primed reverse transcription (TPRT) [12]. ORF0, characterized in humans and chimps, comprises 5′ outbound transcripts encoding potentially intriguing peptides involved in L1 mobility, although their function is unknown [13].

Of note, although the human and mouse genomes contain a high number of L1 copies, accumulating evidence indicates that only around 100 copies and 3000 copies, respectively, are capable of retrotransposition [14–16]. *Short interspersed nuclear elements* (*SINE*s) are another order of retrotransposons. As *SINE* elements utilize *L1*-encoded machinery to complete their propagation, they are usually called non‐autonomous retrotransposons. In humans, active *SINE*s include *Alu* and *SINE‐R‐VNTR*‐*Alu* (*SVA*) retrotransposons [17–19], active *SINE*s in mice contain the 7SL‐derived B1 elements and the tRNA‐derived B2 elements [20]. While *LTR*s and *LINE*s are transcribed by RNA polymerase II [4, 21], transcription of *SINE*s depend on polymerase III [22].

Retrotransposons may be a source of mutations leading to genomic instability and devastating consequences for the host [23]. Therefore, host genomes have evolved several mechanisms to restrict retrotransposition. Interferon-stimulated factors restrain the retrotransposon pre-integration step through post-translational repression [24]. Hosts also rely on several transcriptional repression strategies: locus-specific deposition of repressive chromatin marks [25], like DNA methylation [26, 27] and histone H3K9 trimethylation [28, 29], small RNAs [30, 31], motif-specific protein repressors like KRAB-ZFP/KAP1 module [32–34], which have been extensively discussed elsewhere [35]. Of note, the Human Silencing Hub (HUSH) complex is required for epigenetic repression of young *LINE-1* elements [36–38]. However, in certain conditions, retrotransposons escape from the multiple layers of host surveillance. This escape may result in new insertion events disrupting normal gene function with dramatic consequences and cytotoxic accumulation of TE intermediates. Deregulated TE activity has been linked to cancer [39], neurological disorders [40], male sterility [41] or fetal oocyte attrition (FOA) [42]. However, TE-induced pathologies are not only caused by retrotransposition, but may be triggered by chimeric transcripts [43], *ERV*-derived enhancers [44] and *ERV*-encoded proteins accumulation [45], or a TE-derived cDNA-induced interferon response [46, 47]. The latter plays an important role in defense mechanisms but also in chronic inflammation associated with neurodegenerative pathologies, which were reviewed extensively elsewhere [48, 49].

## Retrotransposons as *cis*-regulatory elements

Several decades ago, Britten and Davidson proposed the "gene battery" theory in which repetitive sequences can be used as a pool to deposit regulatory sequences in specific genomic positions to bring multiple genes under the same regulatory network [50, 51]. By now, the non-random distribution of repetitive elements has been demonstrated extensively and we know that TEs can barcode genes with distinct functions, dictating the time and level of their expression during development by providing regulatory sequences and/or sequestering their associated genes into distinct nuclear domains [52]. It is now becoming clear that retrotransposon integration sites can spread specific functions in a selective evolutionary manner [53, 54]. Indeed, several lines of evidence demonstrate that retroelements can modulate the expression of proximal or distal target genes by acting as a *cis*-regulatory element [44, 55], as cryptic splice sites, or as polyadenylation sites [56–60].

**Fig. 1:**
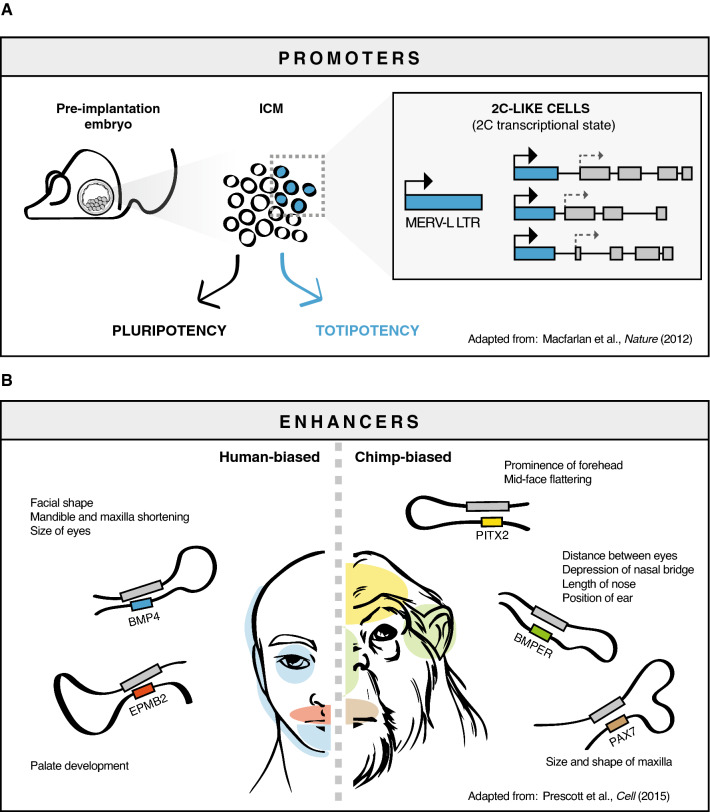
Retrotransposons as cis-regulatory elements. **a** Example of transposable elements functioning as alternative promoters in 2C-like ES cells. Embryonic stem (ES) cells, the pluripotent cells derived from the inner cell mass of the blastocyst, are able to differentiate into all embryo tissues. However, a subpopulation of ES cells can be distinguished for its ability to differentiate into both embryo and extra-embryonic tissues (totipotency). These cells recapitulate the same transcriptional state of totipotent 2 cells, where *MERV* elements are transcriptionally active and work as alternative promoters for two-cell stage specific genes. **b** Example of transposable elements functioning as enhancers. *ERV* and *L1* enriched enhancers in cranial neural crest cells (CNCC) affect the expression of genes involved in craniofacial development. Divergent enhancers between human and chimp contribute to species-specific facial phenotypes. The differential activity of these species-biased enhancers also affects intra-human facial variation

Among *LTR*s co-opted by host genomes, e.g., *MT-C* [61] and the primate-specific *MLT2B3 LTR* [62], most act as promoters. For example, during mouse early development, many transcripts produced at the 2-cell stage are initiated from *MERVL* derived *LTR*, suggesting that this co-opted sequence has played a leading role in cell-fate regulation in placental mammals [63, 64] (Fig. 1a). In human neuronal progenitor cells, epigenetic de-repression of evolutionarily younger *L1* provides alternative promoters for many neuronal protein-coding genes [26].

Enhancers act as central lineage-specific players in orchestrating complex gene expression program underlying developmental and tissue-specific transcriptional programs [65, 66]. One example is represented by the eutherian specific *MER20* elements that integrate information to regulate pregnancy-related gene expression in endometrial stromal cells in placental mammals [67]. Interestingly, retroelements have been shown to contribute to enhancer-mediated craniofacial morphological variation in primate evolution [68] (Fig. 1b).

Identification and functional characterization of enhancers elucidate several common features, including unique epigenomic signature, ncRNA transcription, and TF binding sites [69–72]. Comprehensive common TF binding motif analysis and epigenomic modification profile dissection have shown that retrotransposons exhibit similar enhancer signatures [73–78]. For example, *RLTR13D6/RLTR9* and *RLTR13D5/RLTR13B* family elements acquire enhancer features in embryonic stem cells (ESCs) and trophoblast stem cells (TSCs), respectively. However, only a minor dysfunctional effect on transcription was observed when these elements were perturbed by CRISPR-mediated transcriptional inhibition [79]. This result exposes a weakness of bioinformatics-based genome-wide correlation analysis and prediction as they do not prove functional aspects of TE-derived enhancers [80]. Therefore, several studies have rigorously tested putative enhancers through gain- or loss-of-function CRISPR-based approaches to determine their enhancer potential. Fuentes et al., systematically perturbed the ~ 700 copies of *HERVK* element *LTR5HS* present in the human genome by combining CARGO (Chimeric Array of gRNA Oligos) with CRISPR technology and showed that activation/silencing of *LTR5HS* consistently modulated the expression profile of hundreds of human genes [81]. Pontis et al., downregulated evolutionarily recent *SVA*, *HERVK*, and *HERVH* TE subgroups (TEENhancers) in naive hESC and demonstrated that these elements markedly influence transcription during human embryonic genome activation (EGA) by acting as stage-specific enhancers [82]. A role for ERVs as *cis*-regulatory elements was also found in the innate immunity response by demonstrating their contribution in shaping the evolution of the IFN-response transcriptional network in human [83]. On the other hand, TE-derived enhancers act in complex regulatory contexts. Their activity intersects additively, synergistically, hierarchically, or competitively with other enhancers embedded in the same regulatory landscape [84].

In addition to immobilized retroelements that modulate transcriptomic homeostasis, our team's recent work has also demonstrated that developmentally regulated de novo insertions in somatic cells contribute to shaping the tissue-specific transcriptional landscape. When mouse fibroblasts were reprogrammed into dopaminergic neurons (iDA), reactivation and retrotransposition of L1 elements occurred. De novo insertions mostly landed near neuronal genes and affected proximal chromatin accessibility, thus creating new transcriptional units, especially of lncRNA [85].

## Novel non-coding RNA derived from retrotransposons

**Fig. 2:**
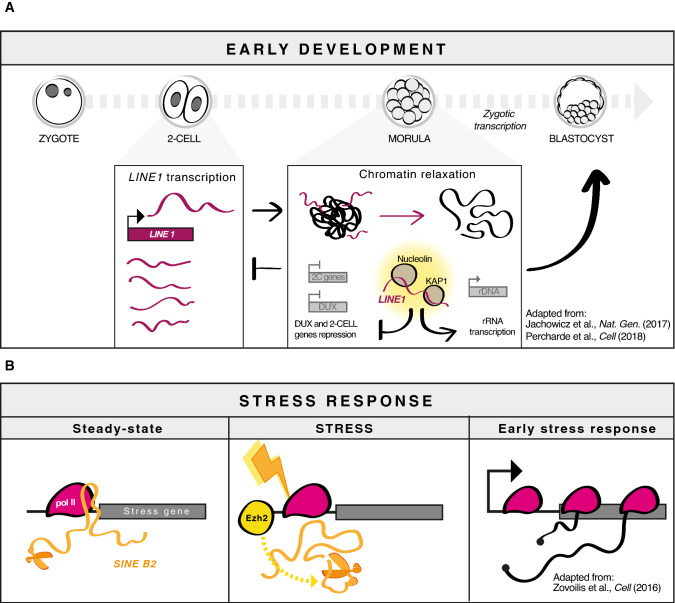
Retrotransposons as non-coding RNA. **a** Example of TE-derived RNA functioning as non-coding RNA during early development. In pre-implantation embryos *L1* are transcriptionally active. *L1* RNA association to chromatin led to global chromatin relaxation. *L1* RNA associates with KAP1 and Nucleolin at rDNA loci inducing their transcription, while repressing DUX and 2C-specific genes to proceed with the development. **b** Example of TE-derived RNA functioning as non-coding RNA in stress response. In resting cells *SINE B2* RNA associates with stress response genes inhibiting RNA Pol II elongation. When cells are stressed the binding of EZH2 to stress loci enhances the self-cleavage activity of the *B2* ribozyme, thus releasing Pol II and allowing transcription of stress response loci

The first evidence for developmental functions of *LINE-1* RNA came from a seminal study from Torres-Padilla's group on early mouse embryogenesis, where they demonstrated how early-stage transcription of *L1* is important for blastocyst formation as it is the key driving force modulating global chromatin structure in *cis* [86, 87]. Nano-CAGE RNA-seq analysis showed TE stage-specific reactivation, with *LINE-1* highly expressed in pre-implantation embryos and downregulated as the differentiation proceeded [87]. Perturbing the tightly regulated level of nuclear *LINE-1* RNA resulted in global chromatin accessibility impairment and reduction of blastocyst formation [86]. Reverse transcriptase activity inhibition did not affect blastocyst formation, and cytoplasmic injection of exogenous *L1* RNA did not revert the effect of nuclear *L1* RNA silencing [86]. These observations strongly suggest a central role for endogenous chromatin associated *L1* transcripts at early development stages in controlling chromatin opening, most probably in *cis*, independent of retrotransposition (Fig. 2a). Of note, the positive effects of *L1* RNA were independent of DNA damage. Percharde et al., dissected the mechanism underlying the role of *L1* RNA in ESC. They demonstrated that *LINE1* RNA acts in *cis* as a nuclear scaffold to facilitate rDNA expression and repression of *DUX*, the master activator of the 2-cell embryo program, thus maintaining ESC self-renewal [88] (Fig. 2a).

In situ analysis of several mammalian cell lines revealed a class of repeat-containing RNAs, mostly *L1*-enriched, broadly and stably associated with euchromatin. These "chromosomal RNAs" are highly stable, accumulate in the nucleus, and remain localized strictly with the interphase chromosome territory in *cis,* where they may help maintain open chromatin structures [89]. In addition to these extensively studied transcripts derived from *L1*, other TE-derived RNAs have been proven to have regulatory functions [90]. *LINE-2* is a source of functional miRNA in the human brain [91], and *Alu* element-containing RNAs (aluRNA) were found to be involved in maintaining nucleolar structure and rRNA synthesis [92]. *HERVH*-derived transcripts provide functional binding sites for a combination of naive pluripotency transcription factors such as LBP9 and OCT4. Their interaction activates hESC-specific alternative and chimeric transcripts, including long non-coding RNAs that modulate pluripotency and maintain human embryonic stem cell identity [93].

RNAs from *SINE B2* repeats in mouse and *SINE Alu* repeats in human, control gene expression by binding RNA polymerase II and suppressing transcription [94–96]. RNAs from these elements have been known for years to be upregulated during the response to various types of cellular stress. Both *SINE Alu* and *B2* RNAs bind and inhibit RNA Pol II [97]. *B2* RNAs and *Alu* RNAs are self-cleaving; upon stress, these RNAs become destabilized and release stalled Pol II to activate stress response genes [98, 99]. Interestingly the cleavage reaction is facilitated by binding to the Polycomb component Ezh2, in this case, an activator [98, 99]. Thus, *SINE* RNAs appear to play a key role in stress response by suppressing or activating transcription based on their processing status (Fig. 2b). In addition to transcriptional regulation, *SINE B2* regulates gene expression post-transcriptionally, by either blocking mRNA nuclear export [100] or by enhancing mRNA translation through the “SINEUP” mechanism [101, 102]. Recent studies report that TEs’ RNA half-life is strongly affected by m6A RNA modification, showing that m6A protects cells from aberrant TE transcript accumulation [103, 104].

## Orchestrating 3D genome architecture as boundaries or compartmentalization factors

**Fig. 3:**
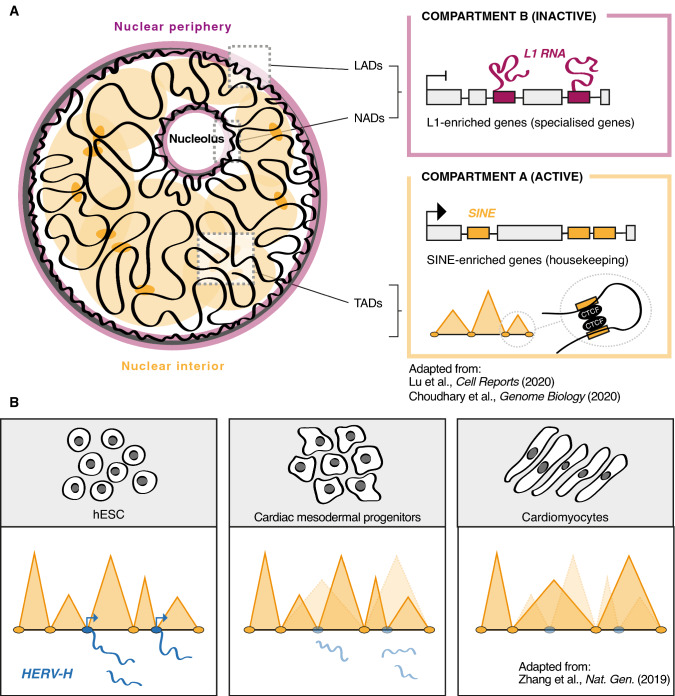
Retrotransposons as 3D genome architecture orchestrators. **a** *L1* RNA associates to *L1* DNA and mediates the sequestration of L1-enriched genes in the inactive compartment (B), at the nuclear periphery, for silencing. *NAD* Nucleolar-Associated Domain, *LAD* Lamina-Associated Domain. Active genes are barcoded by *SINE*s and localized in the nuclear interior (compartment A) where they are actively transcribed. *SINE* elements are enriched for CTCF binding sites, represent TADs (Topologically Associated Domains) boundaries and contribute to chromatin looping. **b** In hESC, transcriptionally active *HERV-H* forms pluripotent specific TAD boundaries which weaken during differentiation to cardiomyocytes. Transcription at *HERV-H* loci is crucial for maintaining chromatin looping at *HERV-H-*dependent TADs

Growing evidence supports a pivotal role for retrotransposons in maintaining higher-order chromatin structure and 3D genome organization in mammals [105].The spatial distribution of *SINE* and *LINE* retroelements has unveiled a differential re-location of those elements into active A compartments at the nucleus interior and inactive B compartments at the periphery of the nucleus (Lamina-associated domains, LADs) and nucleolus (Nucleolus-associated domains, NADs), respectively. This spatial segregation is highly conserved across all eukaryotes and is required to maintain euchromatin/heterochromatin compartmentalization [52, 106–108] (Fig. 3a). Lu et al. recently showed that *L1* RNA preferentially binds to *L1*-enriched genomic loci in ESC by ChIRP-seq [52]. Antisense oligonucleotides (ASO) against *L1* RNA triggered a dramatic re-location of *L1* enriched chromosomal regions from the nucleolus and inactive LADs to the nuclear interior and, in turn, their de-repression [52]. Intriguingly, one recent study highlights a novel mechanism where *L1* DNA and RNA induce HP1α phase separation promoting heterochromatin compartmentalization [109]. These data indicate a role for *L1* RNA in nuclear organization, where *L1* RNA binding to *L1* DNA silences *L1*-enriched genes by directing genomic regions to inactive compartments (Fig. 3a).

Retrotransposons act as either boundary elements or docking sites to facilitate folding and compartmentalization of the genome, whose CTCF binding site bordered topologically associated domains (TADs) are important 3D units. Using Capture-4Tran and 4Tran-PCR, Raviram et al. discovered several young mouse ERV families (*IAPEz*, ETnERV, *RLTR6*, and *MuLV-int*/*RLTR*) engaged in long-range intra-chromosomal interaction and looping formation in specific chromatin compartments [107]. In hESC, transcriptionally active *HERV-H* form TAD boundaries that are specific to pluripotent stem cells and their frequency weaken during differentiation to cardiomyocites [107] (Fig. 3b). Indeed, Hi-C and ChIA-PET have shown that retrotransposon sequences are important anchoring sites for CTCF loops [110]. In particular, *SINE* elements are highly enriched for CTCF binding and over-represented at TAD boundaries [110] (Fig. 3a). Interestingly, CTCF ChIP-seq analysis in six mammalian lineages unveiled a pool of *SINEs* containing CTCF binding sites that are not evolutionary conserved [105]. These data suggest that TE expansion has produced new CTCF binding sites. Thus, altered chromatin architecture and gene regulatory networks may have contributed to the species-specific evolution of mammalian genome structures [74, 111].

However, the pervasive and non-specific distribution of retrotransposons in the genome has raised concerns regarding the functional aspects of co-localization between retroelements and boundary elements, which has been considered a random phenomenon. To establish a functional link between TEs and genome folding, Zhang and Ren investigated a primate-specific *HERV-H* family's ability to create TADs in human pluripotent stem cells [108]. By editing endogenous *HERV-H* sequences, they proved that *HERV-H* elements are essential TADs boundary elements. As further evidence, PiggyBac assisted insertion of *HERV-H* induces de novo organization of TADs at acceptor loci [108].

## New methods to study retrotransposition-independent functions of retrotransposons

In the last decade, advances in the field have shed some light on retrotransposons' role and dynamics during early embryo development, cell differentiation, and specific pathological circumstances. Genomic and epigenomic genome-wide studies have uncovered the importance of retrotransposons in the genome, even if these studies are mainly correlational. However, many questions have not been fully addressed because of the complex nature of repetitive elements. Thus, the improvement and development of new technologies are fundamental to achieving a more in-depth understanding of retrotransposons' functional impact on genome evolution, structure and function, and its mechanisms.

Improved genome editing technologies have allowed modulation of expression/repression of genomic loci, including transposable elements [112–115]. In particular, CRISPR-activation/interference has been used to evaluate *LTR* elements' ability to act as enhancer elements [79, 81]. Church and colleagues have successfully knocked out human *LINE-1* elements using base editors in HEK293T, and human iPS stem cells [116]. As mentioned, there are around 100 and 3000 active *LINE-1* elements in the human (*L1-Hs*) and mouse (*L1-Gf*, -*Tf*, and *-A* family) genomes, respectively [8]. A detailed map of active *LINE-1* loci is fundamental to manipulating these elements' expression through CRISPR-based technologies and testing the effects on genome functions and dynamics.

Interestingly, Ranjan and Gene used a system based on the fusion of an RNA endonuclease with a nuclease-dead Cas9 (dCas9) to target and eliminate toxic microsatellite repeat RNA [117], a transcript produced from a locus that cannot be targeted by an sgRNA with the canonical CRISPRi methods. This approach can be extended to all the other classes of transcripts produced by repetitive or particularly complex loci [117]. RNA editing technology can be applied to follow the dynamics of repetitive RNAs by CRISPR-Cas13-based RNA-labeling with higher specificity than probe hybridization-based probe assays (e.g., fluorescent in-situ hybridization, FISH) [118]. This approach has been used to track lncRNA NEAT1 with a just one sgRNA combined with dCas13b-EGFP [119].

Mounting evidence suggests that retrotransposon-derived transcripts may behave like lncRNAs. Global RNA-DNA/chromatin interaction profiles have been mapped using RNA-centered genome-wide analysis. For instance, *LINE-1* was found to be preferentially bound to *LINE-1* DNA-rich regions of the genome [52]. To understand the function of the thousands of transcripts originated from retrotransposons and other repetitive DNA elements, it is necessary to characterize the RNA–protein interactome to reveal potential regulatory roles of repetitive RNAs in genome structure and gene expression. Towards that end, the improvement of gRNA driven proximal protein labeling assays (e.g., CRISPR-APEX2 technology) [120–122] or RNA pull-down coupled with protein mass spectrometry methods (e.g., ChIRP-MS and RAP-MS) have been instrumental [123–125]. However, immunoprecipitation/pull-down-based assays show several pitfalls when applied to RNAs derived from repetitive sequences or retrotransposons because proper experimental controls and high-specificity probe sets are lacking [126, 127].

Applying these techniques to in vivo studies in animal models or human biopsies is exceptionally challenging because of the limited amount of starting material. The evolution of protein mass spectrometry instruments, like the recent release of 4D (m/z, retention time, ion mobility and signal intensity) proteomics platform, has significantly reduced the peptide detection limit, making it possible to study RNA–protein interactions quantitatively with low input samples [128–130].

Another unresolved and controversial question is whether transcription of retrotransposons or retrotransposon-derived transcripts is a real driver of biological processes [86, 88]. Chromatin-associated lncRNA species enrichment [131] and specific RNA Pol II elongation form precipitation [132] coupled with long-read RNA-seq [133] could address this outstanding question.

## Concluding remarks and future perspective

Initially, retrotransposons were exclusively considered hazardous genetic elements that required strict epigenetic mechanisms for silencing. Now, largely thanks to the development of novel technologies, mounting evidence unveils an essential role of retrotransposon dynamics in fundamental physiological cell functions, from co-option as regulatory elements to orchestrate cell type specific transcriptional programs, chromatin remodeling, 3D genome organization up to immune systems involved in tissue homeostasis and defense mechanisms. A deeper integration of retrotransposons and host genome in which the dynamic nature of mobile elements includes not only DNA but also RNA impacting both nuclear and cytoplasmic functions may be envisaged.

How have these processes evolved? The relatively abundant amounts and quality of ncRNAs produced by these elements in response to environmental signals and the emerging role of RNA in nuclear structure and function, may indicate how these two apparently separated portions of the eukaryotic genome began talking to each other. The two systems could have merged into a unified functional unit, and perhaps further evolved based on epigenome-mediated somatic phenotype variation. As summarized above, retrotransposon-derived RNAs have a strong regulatory potential due to their interaction with proteins involved in chromatin and transcriptional regulation, indicating an exciting new frontier in epigenome biology, developmental processes and control of functional tissue homeostasis. Indeed, finely regulated expression of retrotransposons is essential for proper genome function, as their aberrant expression is strictly correlated to embryo development failure and the onset of several pathologies associated with accelerated aging, cancer, and chronic inflammation.

The development of new technologies, and improvements of the available one, will deepen our understanding of how the host genome balances the detrimental and beneficial effects of retroelements in evolution, development, and pathological conditions.
